# Myopia among Medical Undergraduates of a Medical College: A Descriptive Cross-sectional Study

**DOI:** 10.31729/jnma.8078

**Published:** 2023-03-31

**Authors:** Tina Shrestha, Dilip Kumar Kushwaha, Saurabh Tiwari, Umesh Kumar Sah, Risu Raj, Siddharth Rajak, Anukram Lamsal, Sahasra Joshi, Aliza Dulal, Aashutosh Chaudhary

**Affiliations:** 1 Department of Ophthalmology, Dhulikhel Hospital, Dhulikhel, Kavre, Nepal; 2 Kathmandu University School of Medical Sciences, Dhulikhel, Kavre, Nepal

**Keywords:** *medical students*, *myopia*, *prevalence*

## Abstract

**Introduction:**

Myopia is defined as a refractive error in which rays of light entering the eye parallel to the optic axis are brought to focus in front of the retina when accommodation is relaxed. Globally, myopia prevalence is on the rise for unknown reasons. The aim of the study was to find out the prevalence of myopia among undergraduates of a medical school.

**Methods:**

A descriptive cross-sectional study was conducted among medical undergraduates of a medical school between 2 May 2022 to 3 August 2022 after receiving ethical approval from the Institutional Review Committee of the same institute (Reference number: 21/20). A proforma was distributed among the medical undergraduates and data from known myopic students were collected. A convenience sampling method was used. Point estimate and 95% Confidence Interval were calculated.

**Results:**

Among 279 medical undergraduates, myopia was seen in 119 (42.65%) (36.85-48.45, 95% Confidence Interval). The mean age of the myopic undergraduates was 21±1.47 years.

**Conclusions:**

The prevalence of myopia among undergraduates was found to be lower than the other studies done in similar settings.

## INTRODUCTION

Myopia is defined as a refractive error in which rays of light entering the eye parallel to the optic axis are brought to a focus in front of the retina when accommodation is relaxed.^1^ Globally, myopia prevalence is on the rise for unknown reasons.^2^ The prevalence of myopia is estimated to rise from 28.3% in 2010 to 48.8% in 2050.^2^ This increase in prevalence could have significant social, economic, and educational consequences for society.^3^

Students are considered to have a higher prevalence rate for myopia as longer screen time and study time are associated with the development and progression of myopia.^4^ Myopia is also found to be more prevalent among medical students than in the general population.^5^

The aim of the study was to find out the prevalence of myopia among undergraduates of a medical school.

## METHODS

A descriptive cross-sectional study was conducted among medical undergraduates of Kathmandu University School of Medical Sciences between the study period of 2 May 2022 to 3 August 2022 after receiving ethical approval from the Institutional Review Committee of the same institute (Reference number: 21/20). All the medical students of the institute present during the study period were included in the study. Students who did not provide consent, students who were absent, and students who provided incomplete data were excluded from the study. Convenience sampling was done. The sample size was calculated using the formula:


$$\begin{array}{ll} \text{n} & ={\text{Z}}^{2}\times \frac{\text{p}\times \text{q}}{{\text{e}}^{\text{2}}}\\ & =1.96^{2}\times \frac{0.482\times 0.518}{0.06^{2}}\\ & =267 \end{array}$$

Where,

n = minimum required sample size
Z = 1.96 at 95% Confidence Interval (CI)
p = prevalence of myopia, 48.2%^6^
q = 1-p
e = margin of error, 6%

Hence the calculated minimum required sample size was 267. However, a sample size of 279 was taken for the study.

A proforma was distributed among the medical undergraduates and data from known myopic students were collected. Myopia was classified into low myopia (≤-0.5 and >-6.00 D) and high myopia (≤-6.00 D).^7^ Lighting was considered to be adequate if no artificial lights were required to perform work during the day.

Data were entered and analysed using Microsoft Excel 2013. Point estimate and 95% CI were calculated.

## RESULTS

**Figure 1:**
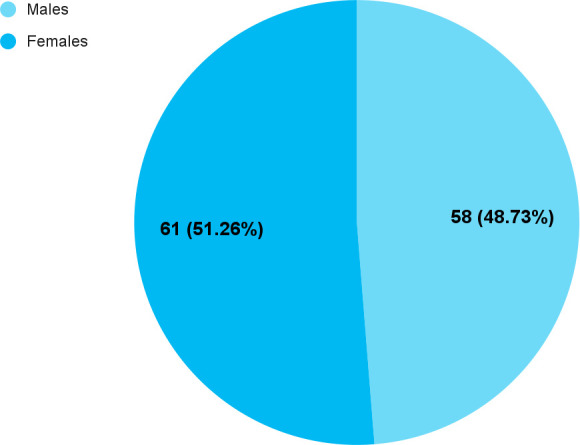
Gender-wise distribution of the students with myopia (n= 119).

Among 279 medical undergraduates, myopia was seen in 119 (42.65%) (36.85-48.45, 95% CI). The mean age of the myopic undergraduates was 21±1.47 years and the range was 18 to 25 years. Out of them, 61 (51.26%) were males and 58 (48.73%) were females (Figure 1).

Out of the undergraduates with myopia, 4 (3.36%) had high myopia (Table 1).

**Table 1:** Classification of myopia among myopic students (n= 119).

Categories	n (%)
Low myopia	115 (96.64)
High myopia	4 (3.36)

Out of myopic students, 79 (66.39%) did not have any parent with myopia, 27 (22.69%) had one parent as myopic, and 13 (10.92%) had both parents myopic. A total of 85 (71.43%) myopic students spent more than 2 hours daily reading for assignments and 101 (84.87%) of the students spent less than 2 hours daily reading for pleasure. A total of 17 (14.29%) myopic undergraduates had a habit of performing eye exercises and 76 (63.87%) had a habit of taking a break after 30 minutes of continuous studying. A total of one hundred two (85.71%) of the students had adequate room lighting (Table 2).

**Table 2:** Characteristics of myopic undergraduates (n= 119).

Characteristics	n (%)
**Daily hours of screen time (hours)**	
None	-
<2	12 (10.08)
2-4	69 (57.99)
>4	38 (31.93)
**Distance of reading (cm)**	
15-25	82 (68.91)
<15	9 (7.56)
≥25	28 (23.53)
**Daily hours of outdoor activity (hours)**	
<2	84 (70.59)
2-3	22 (18.49)
>4	13 (10.92)
**Lighting used to read at night**	
Dim	2 (1.68)
Compact flurescent light (CFL)	29 (24.37)
Light Emitting Diode (LED)	88 (73.95)
**Posture while reading**	
Sitting	114 (95.80)
Sleeping	5 (4.20)
Standing	-
**Hours slept per day (hours)**	
<6	7 (5.88)
6-8	102 (85.72)
>8	10 (8.40)

## DISCUSSION

Myopia can cause a significant burden causing loss of productivity, increasing the cost of health care, decreasing quality of life, and is associated with many ocular comorbidities.^8^ Myopia is correctable using aids such as spectacles or contact lenses and surgery.^9^ The prevalence of myopia varies depending on geography and ethnicity.^8^ East Asian and Southeast Asian countries generally have higher prevalence rates than other parts of the world.^8^ In our study, the prevalence of myopia was 42.65%, among the 279 medical students, which was lower in comparison to prevalence studies done on medical students in our geographic region.^10,11^ In similar studies conducted on medical students, the prevalence of myopia was 64.81%,^10^ in Nepal, 52.78% in India,^11^ and 47% in Pakistan.^12^ The Raine study which was conducted in Australia reported the prevalence of myopia in young adults between the age of 20 to 28 years as 25.8% to 33.2%,^13^ which is lower compared to our study.

The prevalence of myopia is increasing alarmingly in countries of East Asia like China.^8^ In a study conducted on university students in China, myopia was seen in 86.8%.^14^ The high prevalence rate and increasing global prevalence warrant studies to look into the risk factors for the disease. This increase in prevalence is considered to be due to environmental factors like increased near work, decreased outdoor activity, and increased use of electronic devices, along with genetic factors.^2^ In a study, parental myopia was a risk factor while taking breaks after 30 minutes of continuous reading, engaging in outdoor activity, and performing eye exercises were associated with low myopia.^14^

Out of the undergraduates with myopia, 4 (3.36%) had high myopia. The prevalence of high myopia is also estimated to rise from 4% in 2010 to about 10% in 2050.^2^ There is an increased risk of severe and permanent loss of vision from high myopia due to associated comorbidities like retinal detachment, glaucoma, subretinal neovascularization, and thick cataracts.^15^ The global increase in the prevalence of myopia and high myopia may require extensive planning for comprehensive ophthalmologic services equipped with managing and preventing ocular complications associated with high myopia.^2^

Our study had some limitations. As this was a descriptive cross-sectional study, an association between myopia and other factors could not be made. The establishment of causality was also beyond the scope of this research. Also, the small sample size and single-centric nature of the study limit the generalizability of the findings drawn from this study. A study with a higher study design covering a larger geographic range is recommended.

## CONCLUSIONS

The prevalence of myopia among undergraduates of our study was found to be lower when compared to similar studies conducted in similar settings. Higher studies are recommended for the further cause of myopia.
